# Action-perception coupling in violinists

**DOI:** 10.3389/fnhum.2013.00349

**Published:** 2013-07-30

**Authors:** Takafumi Kajihara, Rinus G. Verdonschot, Joseph Sparks, Lauren Stewart

**Affiliations:** ^1^Graduate School of Arts and Sciences, University of TokyoTokyo, Japan; ^2^Rhythm-Based Brain Information Processing Unit, RIKEN BSI-TOYOTA Collaboration CenterSaitama, Japan; ^3^Department of Languages and Cultures, Nagoya UniversityNagoya, Japan; ^4^Department of Psychology, Goldsmiths, University of LondonLondon, UK

**Keywords:** musicians, stroop, audio-motor, learning, violinist

## Abstract

The current study investigates auditory-motor coupling in musically trained participants using a Stroop-type task that required the execution of simple finger sequences according to aurally presented number sequences (e.g., “2,” “4,” “5,” “3,” “1”). Digital remastering was used to manipulate the pitch contour of the number sequences such that they were either congruent or incongruent with respect to the resulting action sequence. Conservatoire-level violinists showed a strong effect of congruency manipulation (increased response time for incongruent vs. congruent trials), in comparison to a control group of non-musicians. In Experiment 2, this paradigm was used to determine whether pedagogical background would influence this effect in a group of young violinists. Suzuki trained violinists differed significantly from those with no musical background, while traditionally trained violinists did not. The findings extend previous research in this area by demonstrating that obligatory audio-motor coupling is directly related to a musicians' expertise on their instrument of study and is influenced by pedagogy.

Professional musicians have been subject to extensive research in recent years, owing to their expertise in sensorimotor mapping. During live performance a concert pianist has to integrate sensory (visual, auditory and somatosensory) with motor information, to bi-manually produce up to 1800 notes per minute whilst monitoring and adjusting for errors (Münte et al., 2002). Theoretical models invoke the use of feedforward control via an inverse internal model, suggesting that, once a certain level of expertise is reached, merely imagining the desired auditory consequences of a performance is sufficient to activate the corresponding motor programmes, and the produced auditory events can be compared with those that were intended, allowing for fast, online error-correction (e.g., Jäncke, 2012). Skilled musicians are thus an ideal model for the study of audio-motor coupling, from both a behavioral and neuroscientific perspective.

Previous studies have used fMRI to compare activation when musicians (a) listened to a piece of music without playing it vs. (b) played a piece of music without auditory feedback (Bangert et al., 2006; Baumann et al., 2007, respectively). Both studies showed areas of overlap between the two conditions, whereby premotor cortex, supplementary motor area and planum temporale were activated in both conditions. Similarly, an fMRI study in violinists, also found auditory activation while participants “played” a known piece silently (by making fingering movements on the chest), this time in right Heschl's gyrus (BA41; primary auditory cortex) as well as left auditory association area (BA42) (Lotze et al., 2003).

A magnetoencephalography (MEG) study demonstrated that merely listening to music which is within the listener's repertoire results in a response within the primary motor cortex (Haueisen and Knösche, 2001). Moreover, a dissociation in the brain surface current density was seen between those notes which would have been played by the thumb and the little finger. The presence of such strong links between the auditory and motor systems is corroborated by transcranial magnetic stimulation (TMS) data from D'Ausilio et al. (2006) who showed increased excitability of motor cortex in pianists listening to a piece they had rehearsed versus a piece with which they were unfamiliar. The instrumental specificity and contextual specificity of such auditory-motor coupling effects is illustrated by a study which compared the brain activation patterns between trumpet players and a musically matched control group of pianists (Gebel et al., 2013). Playing trumpet requires a tight temporal coupling between the fingers and lip muscles, in contrast to playing piano which involves interacting with the instrument through the extremities. These authors used an fMRI compatible trumpet and keypad, and measured activation as participants executed closely analogous movements on both devices, with and without auditory feedback. For the conditions involving trumpet, participants played either with or without using the lips. Playing the trumpet without using the lips and in the absence of auditory feedback resulted in activation of left primary auditory cortex and in the left primary somatosensory lip area. These activations were not seen when trumpet players played on the keypad, nor when pianists played on the trumpet (closely analogous tasks). These findings demonstrate that co-activation of sensorimotor and auditory areas are specific to the context of playing the instrument on which you have been trained.

Several behavioral studies have also addressed the extent to which this coupling between the auditory and motor systems is automatic. For instance, a pair of studies by Drost et al. (2005) demonstrated effects on response time and, in certain cases, errors, when the association between actions and their perceptual effects was manipulated: Pianists were slower to play a musical chord or two note sequences (indicated via various types of visually presented imperative stimuli) when hearing a chord that was incongruent with the required response. While such results suggested that the auditory-motor coupling seen in pianists is likely to be pre-attentive, subsequent findings concerning pitch/space compatibility effects provided a potential alternative explanation for these musician/non-musician differences. Rusconi et al. (2006) and Lidji et al. (2007) independently showed that the mental representation of pitch has a spatially horizontal, as well as vertical, component. With respect to the horizontal component, responses are facilitated for pairings of low pitch with left response location and high pitch with right response location. With respect to the vertical component, responses are facilitated for pairings of low pitch with low response location and high pitch with high response location. These effects have been termed the Spatial Musical Association of Response Codes (SMARC) (Rusconi et al., 2006) or the Spatial Pitch Association of Response Codes (SPARC) (Lidji et al., 2007) and, importantly, have been found to differ in strength between musicians compared with non-musicians. Such a finding is potentially problematic for the interpretation of Drost et al'.s findings on audio-motor coupling, since, in their study, congruent and incongruent trials differed, not only in the extent to which they conform to the associations that would be learned in the course of piano training, but also in the extent to which they exhibit the SMARC/SPARC mapping of low pitch—leftward response; high pitch—rightward response. Thus, the finding of greater interference in musicians compared with non-musicians could potentially be explained on the basis of pitch/space compatibility effects, rather than purely as a function of audio-motor mappings acquired during piano training.

In light of this issue, a recent paper by Stewart et al. (2013) revisited the question of audio-motor coupling in pianists, using a paradigm that was able to pit learned audio-motor mappings against pitch/space compatibility effects. Sequences of numbers were presented aurally at varying pitches which were either congruent or incongruent with the associated finger movements of the right hand. For example, if the numbers 1, 2, 3, 4, 5 were presented at successively higher pitches this would constitute a congruent trial. If the same numbers were presented at successively lower pitches this would constitute an incongruent trial. A second condition expanded the pitch range over which the numbers were heard so as to exceed the range of pitches that could be produced by the hand. In this situation, therefore, the pitch/space mappings would conform to the reported SMARC/SPARC effects but would not conform to the pitch/keypress mappings that had been learned during piano training and the effect of congruency was predicted to be smaller compared with the original condition. The finding of greater interference for pianists (but not non-musicians) in the original condition compared with the “stretched” condition, provides evidence that the results can, at least partly, be attributed to learned pitch-key mappings, as opposed to pitch/space compatibility effects.

To date, much of the literature investigating audio-motor coupling at a behavioral level has examined pianists, but it is important to determine whether previously mentioned findings can be extrapolated to other musician groups, which is a focus of the present study. Violinists constitute an interesting test case, since compared with pianists, this group differs, both in terms of the alignment of the instrument with respect to the body and the orientation of the hand with respect to the instrument. By adapting a violin and devising trials that would be congruent and incongruent with respect to the learned auditory—motor mappings acquired over the course of violin training, we tested the hypothesis that violinists, like pianists, would also show evidence of pre-attentive audio-motor coupling. The biomechanics of violin playing are complex, as noted by Baader et al. (2005), since the production of tones involve combinatorial actions of both limbs and also among left hand fingers (e.g., tone onsets can be produced by finger taps and lifts, depending on the context). Nevertheless, for the purposes of the present study, we simplified the context, restricting ourselves to a range of notes which could be played simply by placing the fingers of the left hand at consecutive positions along the A string.

In addition to determining whether the previously demonstrated interference effects also extend to violinists, we conducted a second study to investigate the impact of teaching method on the strength of these proposed auditory—motor couplings. Although formal musical training in the West prioritizes the mapping between symbol and motor response, developing a strong link between sound and motor response has been argued to be a fundamental musical skill, on which other abilities (sight-reading, playing from memory, performing rehearsed music) can be easily built (McPherson and Gabrielsson, 2002). The Suzuki Method, created by Shinichi Suzuki, is an education system created for Western music, whereby children learn by listening, and only later form a correspondence between symbol and sound. While it has been shown that Suzuki-trained children, in comparison to non-musically trained children show evidence of stronger auditory evoked potentials to violin vs. sine tones (Shahin et al., 2004) to date there has not been any behavioral or neuro-scientific comparison of this group in comparison to traditionally trained students. We hypothesized that children trained via this method would exhibit stronger auditory-motor couplings compared with children trained using a traditional approach where playing from notation is an early focus.

Using the same paradigm as in our first experiment, we compared the interference effects seen in violinists trained using the Suzuki approach to those trained using a traditional approach, and both in comparison to non-trained participants. Given that the approaches of Suzuki training and traditional training converge once music reading is incorporated into the former approach, it was necessary to recruit young musicians, in the early part of their training (up to 5 years). In addition, given the literature on training-induced plasticity, which typically reports correlations between structural changes and training related factors (age of onset; amount of practice), we were careful to match the two musically-trained groups on these variables.

## Experiment 1: Action-perception coupling in violinists vs. non-musicians

### Methods

#### Participants

Two groups of participants were used: 13 violinists (12 females, all right handed, average age 21.92; *SD* = 2.36), recruited from the Royal College of Music in London. The violinists had an average of 16.3 years (*SD* = 2.8) playing experience. Thirteen non-musicians (6 females, all right handed, average age 22.5; *SD* = 4.0) were recruited as a control group. The non-musicians had received no formal musical training. Informed consent was received from each participant prior to the experiment. The participants were not informed about the experimental hypothesis until after the experiment. Each participant was paid £10 for their participation.

#### Materials

A full size violin was modified to record the participants' responses. All the strings were removed and 50 mm light action push-to-make switches were placed at pre-determined positions along the A string (third string from the left). The switches were placed at equal distances representing the notes B5, C5, C#5, and D5 as if being performed on the A string. The switches were connected via 3.5 mm jack inputs to a custom built USB interface that was configured as a Human Interface Device (HID protocol) to capture the participants' responses.

Auditory stimuli were created using a combination of software: (1) REAPER, a digital audio workstation (DAW); (2) celemony melodyne assistant (CMA), a sound modulation tool and (3) independence free (IF), a sound sampler. Since the digits used in the experiment were the index finger, middle finger, ring finger and little finger, the numbers “2,” “3,” “4,” and “5” were used for non-musicians. For violinists, the association between numbers and fingers differs, such that the index finger is referred to as “1,” the middle finger as “2,” and so on. Thus, spoken versions of the numbers “1,” “2,” “3,” and “4” (for violinists) and “2,” “3,” “4,” and “5” (for non-musicians) were recorded using REAPER. The individual samples were then adjusted both visually and by ear so that their apparent onset, as in the moment when the sound could be identified as a word, coincided. This was done to eliminate the chances of participants predicting certain numbers based on initial pauses and to remove timing artifacts that could be introduced if a certain number was identifiable earlier than others. Pitch modulation and pitch drift were normalized and the sound samples were resynthesized to create new samples at particular frequencies using CMA. Each spoken sample was resynthesized at the following frequencies: 493.88, 523.25, 554.37, and 587.33 Hz, which correspond to the pitches B4, C5, C#5, and D5 (Hoenig et al., 2010).

In total thirty-two different sequences were created. Each sequence consisted of five numbers using the number {1, 2, 3, 4} for violinists and {2, 3, 4, 5} for non-musicians. The sequences were randomly generated and always contained one repetition. Example sequences include: 2, 1, 3, 4, 3 (violinist) and 3, 2, 4, 5, 4 (non-musicians). The repetition of one of the numbers was deliberate in order to add an element of ambiguity into the sequences and to prevent participants from predicting which number may appear next. The number sequences for violinist and non-musicians were identical in relation to the fingering pattern.

Sound stimuli for the thirty-two number sequences were constructed from the recorded sound samples for violinists and non-musicians separately using IF. IF was used to trigger the individual samples via a time-coded midi file in order to eliminate onset differences and to minimize any human error through file reuse. Each of the number sequences appeared in two different versions: congruent (low pitch/low number) and incongruent (high pitch/low number). Specifically, in congruent (low pitch/low number) sequences the numbers {1, 2, 3, 4} for violinists and {2, 3, 4, 5} for non-musicians would be represented by the pitches B5, C5, C#5, and D5, respectively. In incongruent (high pitch/low number) sequences the numbers {1, 2, 3, 4} for violinists and {2, 3, 4, 5} for non-musicians would be represented by the pitches D5, C#5, C5, and B5. Within a sequence, each spoken number was 500 ms in duration and there was 1500 ms between consecutive numbers.

### Procedure

Participants were instructed to hold the violin in a natural performing position with their fingers placed over the four switches as follows: index finger on switch one (the switch furthest from their body), middle finger on switch two and so forth. The keys were equal distance from one another so that the switches could be comfortably pressed.

Participants were instructed to think of their fingers in terms of numbers {1, 2, 3, 4} for violinists and {2, 3, 4, 5} for non-musicians and press according to the numbers heard through headphones. Thus, if the number 1 was heard for violinists (2 for non-musicians) the participants would press their index finger, if the number 2 was heard for violinists (3 for non-musicians) the participants would press their middle finger and so on. Participants were informed that the numbers would be presented at different pitches and that they should ignore the pitch and press according to the number heard as quickly and as accurately as possible.

Prior to the experimental trial, each participant performed twenty practice trials in order to become familiar with the finger mapping and instrument of response. The sequences in the practice trial were different to those used in the experimental trial. At the start of the practice trial the interval between each number was 2000 ms. Depending upon a participant's accuracy the interval was increased or decreased accordingly (i.e., staircase thresholding). In the event that a participant performed the sequence correctly, the interval between each number in the subsequent sequence was reduced by 500 ms. In the event that a participant made an error, the interval between each number in the subsequent sequence was increased by 500 ms. In addition, the practice trials ensured that all participants were performing accurately within an interval of 1000 ms between the numbers (500 ms faster than required for experimental trials).

After completing the practice trials, the experiment began. The experimental trials consisted of four blocks of sixteen trials with a 3 s pause between each trial. Participants were instructed to take a short rest between each block and to resume when they were ready. Each block contained both congruent (low pitch/low number) trials and incongruent (high pitch/low number) trials in a pre-determined random order.

### Results

Table 1 shows the response times and performance accuracy for congruent and incongruent conditions in both groups. As can be seen, the response times for the violinists were considerably shorter than those for non-musicians, which was also the case for pianists vs. non-musicians in Stewart et al. (2013). Thus, following the approach taken in our previous paper, we standardized the absolute response times in order to be able to directly compare the effect of the congruency manipulation between violinists vs. non-musicians. This was achieved by computing an RT ratio (incongruent RT/ congruent RT). A comparison of these ratios using an independent-samples *t*-test revealed significantly higher ratios in the violinist group, *t*_(24)_ = 4.2, *p* < 0.001 compared to the non-musician group indicating a larger effect of the congruency manipulation for violinists. Paired *t*-tests were used on the response time data to establish whether each group showed a significant effect of congruency manipulation. This revealed a significant facilitation effect of 41 ms for congruent compared to incongruent trials for the violinist group, *t*_(12)_ = 4.5, *p* < 0.001, and no facilitation for the non-musician group, *t*_(12)_ = 1.4, *p* = 0.147.

**Table 1:** **Mean reaction times (in milliseconds) and mean errors for non-musicians and violinists for both sequence types**.

	**Non-musicians**		**Violinists**	
	**RT (*SD*)**	**Errors (*SD*)**	**RT (*SD*)**	**Errors (*SD*)**
**Congruent**	846 (125)	5.7 (4.6)	700 (115)	7 (6.9)
**Incongruent**	835 (117)	7.9 (9.9)	741 (108)	7 (8.5)
**Effect**	11 (27)	−2.2 (6.4)	−41 (33)	0 (4.3)

#### Error analysis

There was no effect of congruency manipulation, *F*_(1, 24)_ = 1.0, *p* = 0.326, no effect of group, *F*_(1, 24)_ = 0.007, *p* = 0.936, and no interaction between the two, *F*_(1, 24)_ = 1.0, *p* = 0.326.

## Experiment 2: Impact of pedagogical approach on action-perception coupling

### Method

#### Participants

Three groups of children were tested, all within Japan. The first group consisted of 10 children with no musical training (5 females, all right handed, average age 13.8; *SD* = 0.7). One participant from this group was excluded when it became clear that he had preconceived ideas about the hypothesis. The second group consisted of 11 children who had been trained to play violin via the traditional (notation-based) method (7 females, 1 left-handed, average age 13.5; *SD* = 0.8). The third group consisted of 11 children who had been trained to play violin via the Suzuki-based method (6 females, all right-handed, average age 13.5; *SD* = 1.3). This group had received, on average, 5 years of learning by demonstration (*SD* = 2.6). On average, the traditionally-trained children had been playing the violin for 9.5 years (*SD* = 0.8) and the Suzuki-trained children, for 9.9 years (*SD* = 1.5). The amount of time spent practicing for the traditional participants was 111 min a day (*SD* = 81) and 87 min a day (*SD* = 43) for the traditionally-trained and Suzuki-trained children respectively. All the participants provided informed consent and were not informed about the experimental hypothesis until after the experiment.

#### Materials

These were identical to those described in Experiment 1. However, since Japanese people count starting from the index finger (not from the thumb as in most European countries), the numbers {1, 2, 3, 4} were used to construct the sequences, for both groups.

### Procedure

This was identical to that described in Experiment 1: Participants were instructed to think of their fingers in terms of numbers {1, 2, 3, 4} for their left index, middle, ring and little finger, respectively.

### Results

Table 2 shows the response times and performance accuracy for congruent and incongruent conditions in Suzuki-trained violinists, traditionally-trained violinists and non-musicians. As in Experiment 1, there was variability in the overall response times across the three groups so an RT ratio was computed (Incongruent RT/Congruent RT) for each participant. The RT ratios were compared across the three groups using a One-Way ANOVA. This revealed a marginally significant effect of group, *F*_(2, 28)_ = 2.7, *p* = 0.083. A comparison of these ratios using an independent-samples *t*-test revealed higher ratios for Suzuki-trained compared to traditionally-trained violinists, *t*_(12)_ = 1.5, *p* = 0.08 and for Suzuki-trained violinists compared to non-musicians, *t*_(12)_ = 2.0, *p* < 0.05 (both one-tailed). Traditionally-trained violinists did not differ significantly in comparison to the non-musicians, *t*_(17)_ = 1.3, *p* = 0.1. Paired *t*-tests were used on the response time data to establish whether each group showed a significant effect of congruency manipulation. This revealed a significant facilitation effect of 49 ms for the traditionally-trained group, *t*_(10)_ = 2.5, *p* < 0.05, a significant facilitation effect of 136 ms for the Suzuki group, *t*_(10)_ = 2.3, *p* < 0.05, and no facilitation for the non-musician group, *t*_(8)_ = 0.04, *p* = 0.96.

**Table 2:** **Mean reaction times (in milliseconds) and mean errors for traditional, Suzuki and non-musician groups for congruent and incongruent trials (Experiment 2)**.

**Group**	**Congruent**		**Incongruent**		**Effect**	
	**RT (*SD*)**	**Errors (*SD*)**	**RT (*SD*)**	**Errors (*SD*)**	**RT (*SD*)**	**Errors (*SD*)**
Traditional	655 (70)	6.6 (1.7)	704 (107)	8.0 (3.4)	49 (66)	−1.4 (3.1)
Suzuki	669 (119)	7.1 (3.4)	805 (186)	9.1 (4.3)	136 (193)	−2.0 (3.0)
Non-musician	961 (226)	8.6 (5.0)	963 (168)	8.3 (3.4)	1 (92)	0.2 (0.6)

#### Error analysis

There was a main effect of congruency on accuracy, *F*_(1, 28)_ = 4.4, *p* < 0.05. Planned comparisons showed that this was driven by the Suzuki group alone, where significantly more errors were made for incongruent compared to congruent trials, *t*_(10)_ = 2.2, *p* < 0.05.

## General discussion

The aims of the present study were to replicate and expand upon the previous evidence for pre-attentive auditory-motor coupling in musicians by (a) examining a novel group of musicians and (b) investigating the influence of the pedagogical approach used in acquiring instrumental skills (traditional vs. Suzuki-based training). The results of Experiment 1 clearly showed that violinists, but not non-musicians, were affected by the pitch at which an aurally presented sequence was presented. Sequences presented at pitches that were incongruent with respect to the cued motor responses elicited slower responses compared with sequences where pitches and motor responses corresponded. In comparison to the previously reported data from pianists in Stewart et al. (2013), the between group differences were more striking, with non-musicians showing no significant congruent/incongruent differences (while non-musicians in the previous study showed a significant difference, albeit a smaller one compared with the pianist group). This may be because the current paradigm, based around holding a violin in the typical manner, does not involve spatial compatibility effects such as the SMARC/SPARC effect (Rusconi et al., 2006; Lidji et al., 2007) which may have contributed to the observed differences in the previous study.

In addition, the present study also took account of another variable which may have contributed to the previously demonstrated interference effects. The aurally presented sequences in the previous study used a pitch range (“G,” “A,” “B,” “C,” “D”) that conforms to the G major scale. It could be argued that this tonal framework, whereby each pitch corresponds to a particular scale degree, could provide musicians with an additional frame of reference that may influence how they approach the task. In order to avoid this in the current experiment, we used a chromatic scale such that scale degrees could not be used point of reference (Taylor, 2002). The finding of a strong effect of congruency manipulation in the violinist group confirms that the effect demonstrated in the present study cannot be due to the potential use of tonal information as an anchor point.

The results presented here, in combination with the findings of Stewart et al. (2013), challenge the claim made by Drost et al. (2007) that the auditory stimulus involved in an automatic audio-motor coupling must be specific to the instrument that has been trained. Drost et al. (2007) found that when pianists and guitarists were instructed to perform one of two visually presented chords, interference only occurred when the imperative (to-be-ignored) audio-stimuli was a keyboard instrument for pianists and a guitar for guitarists. In the context of the current paradigm, pitch information is carried by the voice rather than the instrument of study (violin here; piano in Stewart et al., 2013), suggesting that pitch *per se* can be a sufficiently strong cue by itself to influence action. Overall, the results of Experiment 1 demonstrate that audio-motor coupling is a learned phenomenon that is directly related to a musicians' expertise on their instrument of study.

The musicians who took part in Experiment 1 had all experienced traditional formal musical training which emphasizes playing from musical notation from the outset. Clearly, these musicians were in possession of strong auditory-motor associations, such that they could not suppress irrelevant pitch information when preparing a motor response. However, it must be borne in mind that these musicians had, on average, 16 years of training, over which time the three way associations between symbol, sound and action can be expected to have become highly ingrained. Experiment 2 investigated whether early training in a method that emphasizes sound to action correspondences would forge stronger automatic auditory-motor mappings compared with a traditional notation-based approach. Thus, Experiment 2 used the same paradigm as described in Experiment 1 to ask whether the effect of congruency manipulation was greater for musicians who had been trained with the Suzuki method, which emphasizes sound to action correspondences, as compared with musicians trained using a traditional approach. The pedagogical differences between these approaches are maximal in the early years of training, before the Suzuki-trained students have been introduced to music notation. Thus, we recruited violinists who had, on average, ~5 years of Suzuki-based training, and used the paradigm in children for the first time. The demonstration of a significant effect of congruency manipulation in both musicians groups (Suzuki and traditionally-trained) demonstrates that automatic audio-motor coupling does not require extensive training (the children in Experiment 2 had 5 years training, on average, compared with 16 years, for the adults in Experiment 1). The between group comparisons were in line with our predictions: violinists who were Suzuki trained (but not traditionally trained) showed a significantly greater effect of congruency manipulation compared with the non-musicians. The Suzuki trained violinists also showed a greater effect compared with the traditionally trained violinists, albeit with marginal significance.

It is of interest to consider the potential underlying neural basis for the audio-motor coupling that we have demonstrated and a study by Lahav (2007) is helpful in this regard. These authors trained non-musicians to listen to pitch sequences and play them back on a piano keyboard, learning by trial and error over a several days. fMRI was used to contrast activation when listening to sequences that they had learned to play, versus similar sequences they had not learned to play. This contrast revealed a fronto-parietal motor-related network (including Broca's area, the premotor region, the intra-parietal sulcus, and the inferior parietal region). This is congruent with the findings that listening to familiar music without playing it and playing a familiar piece without auditory feedback result in a “filling” in of the missing modality (Bangert et al., 2006; Baumann et al., 2007) yet is more striking for the fact that the learning had taken place over days, not years. A similar study which measured activation as learning of audio-motor sequences progressed suggested that learning was accompanied by a reduction in activity within the dorsal auditory action stream (Chen et al., 2012). Analogous structural and functional connectivity studies have yet to be conducted but it can be hypothesized that a training approach that emphasizes sound to action associations would result in earlier and/or more robust physical and functional connections within this pathway. A further possibility is that pedagogical approaches may refine not only the absolute strength of structural and/or functional connections between auditory and motor systems but the manner in which these interactions can be flexibly and dynamically utilized (e.g., Jäncke, 2012). A longitudinal study, preferably with the ability to randomly allocate children to one pedagogical approach or the other would be ideal to address such a question.

In summary, the two experiments presented here build on previous work investigating auditory-motor coupling in musicians. The existence of pre-attentive perception-action links, previously shown in pianists were also seen in trained violinists, as well in a group of children, with considerably less training than professional adult musicians. Within this group of children, the type of musical training experience (traditional vs. Suzuki) impacted upon the strength of auditory-motor coupling, with Suzuki trained musicians exhibiting a larger effect. This has educational implications and suggests that it is not only *what* you learn but *how* you learn it that can have significant consequences on the representations that are formed.

### Conflict of interest statement

The authors declare that the research was conducted in the absence of any commercial or financial relationships that could be construed as a potential conflict of interest.
